# Making invisible chemicals used in plastic materials visible

**DOI:** 10.1016/j.ebiom.2024.105422

**Published:** 2024-11-01

**Authors:** Leonardo Trasande

**Affiliations:** a Department of Pediatrics, New York University Grossman School of Medicine, New York, NY, USA; b Department of Population Health, New York University Grossman School of Medicine, New York, NY, USA; c New York University Wagner School of Public Service, New York, NY, USA

A large body of evidence now supports adverse health effects of exposure to chemicals ubiquitously used in common plastic materials. Attributable biological dysfunction and chronic diseases span the entire lifespan, from prematurity to obesity, cardiovascular disease, reproductive conditions, asthma, atopy and lung function, reduced responses to vaccines, autoimmune conditions, and cancers. In the US, diseases and dysfunctions due to plastic cost 1.2% of its Gross Domestic Product annually.^1^ Additionally, plastic exposures drive racial and ethnic disparities in chronic disease.^2^

Although studies of the risks posed by chemicals used in plastic have focused on a small subset of the 16,000 that have been identified,^3^ findings from the laboratory have been largely confirmed in humans, and independently corroborated by independent scientific entities. These include but are not limited to the Endocrine Society, the World Health Organization and United Nations Environment Programme, the International Federation of Gynecologists and Obstetricians, the American Academy of Pediatrics, and the World Obesity Federation.^4^ The evidence of adverse effects is strongest for phthalates which are used to soften plastic, most especially polyvinyl chloride-based plastics; bisphenols, which are monomers of polycarbonate plastics used in aluminum can linings and thermal paper receipts; per- and polyfluoroalkyl substances (PFAS) used in fluoropolymer plastics; and brominated flame retardant additives.

If you ask a member of the general public about plastic, the focus of that conversation is likely to be on micro- and nanoplastics (MNP) rather than harmful chemicals. MNPs have been detected in nearly every organ of the human body.^5^ We know MNPs may either cause direct injury to tissue, akin to that induced by outdoor air pollutants, or carry plastic monomers (such as bisphenols), contaminants (e.g. metals), non-intentionally added substances, and additives, such as phthalates. The quantity of microplastics in carotid endarterectomy samples has been associated with a fourfold increase in the composite event of heart attack, stroke and death.^6^ But we do not yet have standardised methods to quantify MNPs, and heterogeneity in utilised methods challenge the ability of researchers to compare results across studies.^7^ We have enough evidence now to act to reduce plastic exposures, even if the effects are due to the invisible.

Preventing plastic exposure is very much possible. A dietary intervention rapidly reduced phthalate and bisphenol exposures, as did selecting phthalates and fragrance-free personal care products.^8^ Notably, interventions have been successful in low-as well as high-income populations. “Healthy building” interventions have reduced levels of dust substantially, and in relationship to the intensity of the modifications. Insofar as interventions are sustained, the opportunity for reductions in chronic disease is real.^9^

## Past barriers to laboratory testing for chemicals used in plastics

If the science documenting health hazards is particularly robust regarding chemicals used in plastic (especially compared to MNPs), why aren't clinicians more curious about the invisible chemicals in plastic? Among the concerns raised about quantifying concentrations of phthalates, PFAS, bisphenols and other chemicals of concern is that the results would not change preventive guidance or other aspects of health care delivery. Although it is true that many of the suggested steps to reduce plastic exposure would not change based upon quantified concentrations, exposure patterns from biomonitoring data could reveal sources of greater concern (e.g., lower molecular weight phthalates used in cosmetics). More salient concerns include cost (currently $300–500 per assay depending upon laboratory, scope of testing, and limits of detection) and accessibility of testing. The latter barrier has been lowered with the growth of high-performance liquid chromatography and tandem mass spectrometry in clinical laboratories over the past 20 years.

Is the health-care provider the barrier? Health-care providers have been shown to have low self-efficacy and knowledge in managing environmental hazards.^8^ It is possible that a default reaction in a busy clinic is to dismiss concerns around preventing toxic exposures and to refocus on treatment instead. To be fair to healthcare providers, the science of plastics affecting human health has largely emerged in the past 15 years. However, that argument does not excuse the need for a change in paradigm, or the necessity of incorporation of plastic-related chemical hazards in undergraduate, graduate and continuing medical education. Knowledge and communication about these hazards are not solely the province of primary care, as these chemicals have been identified to contribute to both the onset and progression of chronic diseases.

## PFAS testing: the national academy of sciences, engineering and medicine (NASEM) report

Prompted by widespread water contamination of these chemicals, and a large body of human evidence for harm, in 2022, NASEM recommended that the Centers for Disease Control and Prevention (CDC) update its clinical guidance to offer PFAS testing to patients likely to have a history of elevated exposure (defined as occupational exposure or living in areas where regular consumption of water could reasonably produce health effects). NASEM also suggested different levels of medical monitoring based upon measurements of six PFAS most commonly identified in laboratory assays in serum. For serum levels of the sum of six PFAS between 2 and 20 ng/mL, additional lipid screening in adults and monitoring of hypertension in pregnancy are recommended. Whereas, if levels exceed 20 ng/mL, thyroid hormone measurements, urinalysis and screening for testicular cancer are indicated.

This watershed report represents the first expansion of environmental biomonitoring beyond lead for decades. It acknowledges the need for biomonitoring capacity to be developed in clinical laboratories. The CDC suggests testing by Clinical Laboratory Improvement Amendments certified laboratories, but NASEM suggests instead the need for laboratories to have strong quality assurance and quality control programs, to use US National Institute of Standards and Technology standard reference materials, and to achieve limits of detection and proficiency in line with those produced by CDC in the National Health and Nutrition Examination Surveys.

## Mainstreaming chemical testing: a way forward

The NASEM report and CDC guidelines have rapidly transformed the landscape of clinical care for populations exposed to chemicals used in plastics. Testing is becoming more commonplace given that it is now an insurance option. New Hampshire (US) requires insurance companies to cover PFAS testing, and other US states are likely to follow suit.

When will routine testing for phthalates, bisphenols and flame retardants in the clinic follow? We first need to address clinical laboratory capacity and empower health-care providers with knowledge. In conjunction with this, we should assess the impact of testing on behavioural change and, ultimately, levels of exposure. Some researchers have argued that the testing could produce adverse effects on vulnerable subpopulations, causing dietary changes that may adversely affect health in other ways. Although this potential should be thoroughly investigated, interventions to reduce plastic exposure, such as increased consumption of fresh fruits and vegetables, align largely with overall efforts to reduce cardiovascular risks. Importantly, the consumption of highly processed food has been directly associated with phthalate exposure.^10^ Given the documented role of phthalates in obesity and cardiometabolic risks,^1^^,^^4^ phthalate exposure may be another explanatory factor for the adverse risks of highly processed food, independent of the nutrient profile most often raised as the driver of associated health issues.

Cost remains an important concern. Can scaling up testing capabilities reduce the cost of testing or should more cost-efficient methods be developed? There is kinship here to the advances in multiomics that provide opportunities for genetics, epigenetics, metabolomics and proteomics to transform health care delivery. Costs of multiomic testing and a lack of guidance for clinicians to interpret results have limited the incorporation of genetic and epigenetic testing. Quantification of chemicals used in plastic in human samples should be fully incorporated as part of an exposomic framework, first described by Wild 20 years ago.

Medical societies such as the Endocrine Society, International Federation of Gynecologists and American Academy of Pediatrics have shown great leadership in documenting effects of chemicals used in plastic. The time has come to take a further step forward in incorporating testing as part of clinical care. Health-care providers need to accept that the seemingly invisible presence and burden of chemicals in plastics is, in reality, visible through chronic illnesses that are increasing as plastic consumption continues to grow. Whether or not testing is covered by health insurance, interventions to reduce exposure should become part of the broader primary, secondary and tertiary prevention mindset. Furthermore, exposure related to health care is a part of the problem, particularly with the widespread use of plastic in medical devices and equipment, which are known to contribute phthalate and bisphenol exposures. Not all of this plastic use is truly essential, and much of it could be avoided, ultimately reducing related adverse health effects.

Plastic production and consumption are increasingly being shifted from high-income countries into low-income and middle-income countries, along with their associated harms. Plastic waste is already visible in industrialising countries, where waste picking is increasingly a form of subsistence. The United Nations has set on a course to negotiate a Global Plastic Treaty to address environmental and health challenges posed by the explosion in plastic production. Only through a serious and concerted effort to turn off the plastic tap will we apply the lessons learned from plastic use (largely identified in industrialised countries) and avoid the invisible chemicals used in plastic materials from becoming visible in the form of a larger global pandemic of chronic disease in low-income and middle-income countries. Testing, which is more widely available in high-income countries, will be needed globally to monitor progress and document the impact of the steps taken to stem the tide of the plastic crisis.

After many years of invisible increases in temperature, we now see the climate crisis visibly in the form of increased hurricanes and storms, heat waves and vector-borne illness outbreaks. This has deep parallels to the plastic pollution crisis, which has only become visible through the recognition of both microplastics in human tissues as well as the health risks posed by MNPs and chemicals in plastics. Adding to the commonalities between the two phenomena and the dangers they pose, plastic is generated through fossil fuel consumption. Crude oil is used to make polyethylene plastics, while natural gas is used to make the building blocks for polypropylene. Plastic production is increasingly producing climate change.

Looking to the next ten years ahead, biomonitoring initiatives are needed to visualise this issue, particularly in low-income and middle-income countries. We have substantially advanced our understanding of the health effects of plastic production and consumption over the past decade. Much as we have progressed in combating climate change by making it visible through regular measurement of its presence and effects, we can achieve substantial improvements in human health by making the chemicals used in plastic materials similarly more visible. The technology to measure MNPs will eventually advance and make concerns even more apparent, but the threat of the invisible requires us to act now.

## Contributors

LT is the sole author.

## Declaration of interests

Leonardo Trasande, MD. MPP has received royalties or licenses from Houghton Mifflin Harcourt, Audible, Paidos and Kobunsha, unrelated to the present work. He has received support for travel or meetings from the Endocrine Society, World Health Organization, the United Nations Environment Programme, Japan Environment and Health Ministries, and the American Academy of Pediatrics, unrelated to the present work. He has served as an unpaid member of the Scientific Advisory Board of Beautycounter, Ahimsa, and Grassroots Environmental Education and Footprint, unrelated to the present work.

## References

[1] Trasande L., Krithivasan R., Park K., Obsekov V., Belliveau M. (2024). Chemicals used in plastic materials: an estimate of the attributable disease burden and costs in the United States. J Endocr Soc.

[2] Attina T.M., Malits J., Naidu M., Trasande L. (2019). Racial/ethnic disparities in disease burden and costs related to exposure to endocrine-disrupting chemicals in the United States: an exploratory analysis. J Clin Epidemiol.

[3] Wagner M., Monclús L., Arp H.P. (2024). State of the science on plastic chemicals-Identifying and addressing chemicals and polymers of concern. Zenodo.

[4] Kahn L.G., Philippat C., Nakayama S.F., Slama R., Trasande L. (2020). Endocrine-disrupting chemicals: implications for human health. Lancet Diabetes Endocrinol.

[5] Thompson R.C., Courtene-Jones W., Boucher J., Pahl S., Raubenheimer K., Koelmans A.A. (2024). Twenty years of microplastics pollution research—what have we learned?. Science.

[6] Marfella R., Prattichizzo F., Sardu C. (2024). Microplastics and nanoplastics in atheromas and cardiovascular events. N Engl J Med.

[7] Cui T., Shi W., Wang H., Lihui A.N. (2022). Standardizing microplastics used for establishing recovery efficiency when assessing microplastics in environmental samples. Sci Total Environ.

[8] Harley K.G., Kogut K., Madrigal D.S. (2016). Reducing phthalate, paraben, and phenol exposure from personal care products in adolescent girls: findings from the HERMOSA intervention study. Environ Health Perspect.

[9] Trasande L. (2024). The role of plastics in allergy, immunology, and human health: what the clinician needs to know and can do about it. Ann Allergy Asthma Immunol.

[10] Baker B.H., Melough M.M., Paquette A.G. (2024). Ultra-processed and fast food consumption, exposure to phthalates during pregnancy, and socioeconomic disparities in phthalate exposures. Environ Int.

